# Illuminating the “black box” of complex suicide prevention interventions: towards a theory of implementation using the normalisation process theory

**DOI:** 10.3389/frhs.2025.1473682

**Published:** 2025-08-07

**Authors:** Sadhvi Krishnamoorthy, Gregory Armstrong, Sharna Mathieu, Victoria Ross, Kairi Kõlves

**Affiliations:** ^1^ Australian Institute for Suicide Research and Prevention, World Health Organization Collaborating Centre for Research and Training in Suicide Prevention, School of Applied Psychology, Griffith University, Brisbane, QLD, Australia; ^2^ Centre for Mental Health and Community Wellbeing, The University of Melbourne, Carlton, VIC, Australia

**Keywords:** mechanisms, theory-informed, implementation science, suicide prevention, complex intervention, context, normalisation process theory

## Abstract

**Introduction:**

Although there has been some investigation into what works in suicide prevention, exploration into the mechanisms by which implementation strategies impact outcomes or how and why strategies work remains largely under-studied. Consequently, implementation efforts often lack a clear strategy, and may contribute little toward the desired outcomes. This study aims to explore and examine the role of context and mechanisms involved in the implementation of complex suicide prevention interventions.

**Methods and materials:**

In-depth qualitative interviews were used to explore relevant stakeholder experiences of implementing complex suicide prevention interventions. Stakeholders (nine intervention leaders, five implementors and two lived experience advocates) from six interventions were purposively recruited for their experiences involved in implementing complex interventions in real-world settings. The Normalisation Process Theory translational coding manual was used to map data related to the primary and secondary constructs defined in the theory and its extensions.

**Results:**

Three domains pertaining to implementation context, mechanisms, and outcomes (CMO) were explored. Participants expressed their agency by: using contextual influences to modify the design and plan for delivery; making adaptations to the form and function of interventions and characteristics of the implementation environment to suit intervention needs; and, leveraging the intervention environment to integrate the intervention into existing systems and practices. Activities and strategies served multiple mechanisms involved in: understanding what the work entails; who does the work; developing the capacity to implement the work; and, understanding the work. An interdependent and interacting relationship between mechanisms emerged. Outcomes related to: change in existing practices; the ways in which people are organised; changes in existing norms; and, incorporation of the intervention into daily practice were observed.

**Conclusion:**

This study is notable in its exploration of mechanisms underlying implementation of complex suicide prevention interventions. Data from this study can help inform the development, refinement and use of specific implementation strategies and understand the applicability of strategies across varied contexts. The study also demonstrates the use of an implementation theory to inform practice and potentially contributes to an understanding of what works, why, for whom and in what context. There is a need for a paradigm shift towards the use of more theory based and informed approaches to understand causal links between implementation strategies, context, mechanisms, and outcomes.

## 1 Introduction

Although implementation processes are complex and emergent, they are rarely arbitrary—May and Finch [(1), p. 548].

In 2001, the Institute of Medicine observed a *quality chasm* characterised by a substantial gap between care that could be delivered based on scientific knowledge and the care that is delivered in practice (2). More than two decades later, this evidence-practice gap continues to prevail across the scientific continuum. We know that it takes about 17 years for evidence-based practice to be utilised in routine care (3). Once the benefits of a program or practice are established, important real-world concerns such as improving outcomes for the broader community, achieving a return on investment in our research endeavours and preventing wasteful expenditure of resources in the production and reporting of research evidence, is crucial (4, 5). Implementation science has evolved to address some of these needs and priorities.

Implementation science aims to promote the adoption and integration of evidence-based practices, interventions, policies into routine care settings to improve population health (6). However, successful delivery and adoption of evidence-based practices is influenced by various determinants across multiple levels, including intervention characteristics, interpersonal factors, and organizational/systems factors (7). Therefore, the discipline concerns itself with identifying and prioritizing these determinants to inform the selection and application of strategies aimed at promoting implementation (8) and explaining the causal mechanisms involved in delivery and adoption of an intervention (9). Eventually, successful implementation can lead to effective practice and also shape attitudes, foster widespread adoption, and ensure sustainability (10).

Suicide is complex and does not result from a single causal factor or event. Due to the complexity of factors, adopting a coordinated, comprehensive, multisectoral approach involving health care, education, employment, social welfare, justice, agriculture, nongovernmental organizations, community organizations and others has been emphasised (11, 12). Unfortunately, a care gap exists such that new developments are infrequently translated into practices, programs, and policies for saving people's lives (13). Although, the evidence around what may help reduce suicides is gradually growing (14), it is far from convincing. Achieving a thorough and robust evaluation to understand how evidence translation occurs, involves logistical and methodological challenges such as: (a) developing generalisable suicide prevention metrics as well as aligning process and outcome measures for comparisons across systems (15); (b) inappropriateness of the randomised controlled trial (RCT) design to evaluate multilevel and universal interventions (16); and (c) reliance on traditional approaches to evaluation which may limit opportunities for learning about the intricate pathways between the program (as a whole and via its component parts) and intended outcome(s) (66). Broadening the scope and meaning of program evaluation (16), with a focus on structures, processes, inputs, and resources has been recommended (17).

Applying evidence-based interventions within real-world multilevel settings is complex because strategies need to be multifaceted and reflect context needs (18). From a complexity lens, interventions and contexts are co-evolving organisms and need to be seen as dyads (19). Complex interventions, such as those undertaken for evidence translation, may therefore aim to change the ways people think, act, and organize themselves, or may instigate a process with the goal of generating a new outcome (20). This means that traditional approaches to examining such interventions (for example, with a focus on effectiveness) may oversimplify a very complex reality. Hawe (21) argues that we have moved away from viewing interventions as simply programs, technology, or a set of products to viewing them as relationships, resources, power structures, symbols, and a set of values.

A focus on the effects and evaluative conclusions drawn from a social program instead of *how* the effects are produced was first defined as the “black box” evaluation (22). Social programs and policies embody theory in practice, and exploring mechanisms involved in program theory holds significant potential in unpacking this black box (23). Examining mechanisms as part of theory based or theory-driven approaches, strengthens our understanding of how and why programs work, with whom, and under what circumstances (24). However, mechanisms are often conflated with program activity and/or intervening variables. Astbury and Leeuw (23) define mechanisms as—“underlying entities, processes, or structures which operate in particular contexts to generate outcomes of interest” (p. 368); and suggest that they are—(a) usually hidden; (b) sensitive to variations in context; and (c) generate outcomes. Programs based within social settings not only comprise tangible elements (inputs, activities, and outcomes) but also an interplay between mechanisms and the context which shape our observations (25).

Examining mechanisms underlying complex, multilevel suicide prevention interventions could provide insights into how evidence-practice translation occurs. The current study aims to examine *how* and *why* complex suicide prevention interventions *work* using the Normalisation Process Theory (NPT). The NPT (1, 26) is a theory of implementation that focuses on what people (individuals and groups) do to effect change; in other words, the social organisation of work. According to the theory, “implementation processes are…organized and organizing expressions of human agency that involve patterns of dynamic and contingent interactions within a specific context, over time” (1, p. 540). Hence, to understand *what it is about the program that works*, it is important to explore *how* people do their work. The current study utilises relevant constructs from the iterative development of NPT representing the *mechanisms* that motivate and shape implementation processes, the *outcomes* of these processes and the *contexts* in which these are operationalised (27).

We aim to answer the following questions:

• How do contexts influence complex suicide prevention interventions and their implementation environment?
• How do agents (stakeholders involved) make sense of, commit to, execute, and evaluate complex suicide prevention interventions?
• What shifts in practices, organizational structures, interpersonal dynamics, and norms, can be observed as complex suicide prevention interventions are implemented, evolved, and integrated into various settings over time?

## 2 Methods and materials

### 2.1 Study design

A qualitative study design was adopted to explore participant stakeholder experiences of implementing complex suicide prevention interventions. Such a design is not only important for exploring the influence of the context in which implementation occurs and processes involved in delivery, but also to gather data to theorise about relationships between different factors impacting implementation processes (28). A generic qualitative approach was used to uncover reflections related to the implementation environment, processes, and outcomes (29). The project was approved by the Griffith University Human Research Ethics Committee (GU ref no: 2022/286).

### 2.2 Intervention characteristics

The study aimed to examine the aggregate of experiences related to implementing complex suicide prevention interventions to understand how and why they work (or not) within real-world settings. Interventions were considered complex if they were *multilevel* and *multicomponent.* For the purpose of this study, interventions were considered *multilevel* if they were implemented across at least two levels of a social-ecology (e.g., implemented at the individual level and at the organisational level within a health care setting) and/or levels of prevention (e.g., a universal intervention such as restricting access to means and a selective intervention such as gatekeeper training for community stakeholders). To be considered multicomponent, interventions needed to be comprised of three or more distinct intervention components (e.g., school mental health programs, educational workshops, media campaigns) (30). Other factors contributing to complexity such as the diverse recipients or targets of intervention activities (target populations), by whom the intervention was delivered and the context of delivery (31) were also considered as contributing to complexity. Interventions were identified based on a comprehensive systematic review on utilisation and application of implementation science within complex suicide prevention interventions (32).

### 2.3 Participants

#### 2.3.1 Sampling

Once a pool of eligible interventions was identified through the aforementioned systematic review, participants were purposively sampled for their diverse experiences involved in leading, delivering, and guiding the implementation of complex interventions (33). Participants involved at different levels of program delivery were identified to explore different vantage points on the delivery and adoption of these interventions (34). Geographical spread and representation from high income (HIC) and low-middle income countries (LMICs) was also considered.

Once identified, email invitations to leaders of complex suicide preventions were sent leveraging existing networks of a team member (KK, PhD in Sociology), a senior researcher with over 20 years of experience in the sector. This was an important step for supporting recruitment to the study. A respondent driven or snowball sampling technique was also used to identify participants with other roles and responsibilities involved in intervention delivery.

#### 2.3.2 Sample characteristics

Participants (key stakeholders) belonged to one or more of the following categories: (a) leaders or principal and chief investigators, project directors, senior research fellows, individuals who have had experience of leading complex suicide prevention interventions; (b) project team members, project managers, day to day implementation practitioners—individuals who have had the responsibility of ensuring interventions are implemented as per protocol; (c) lived experience advocates or persons with lived experience of suicide who use their lived experience for formal or informal activity prompting and supporting change in the suicide research and prevention sector.

Leaders were identified for their broad perspectives on intervention design, overall approach, and delivery; project team members (or implementors) were identified for their experiences of day-to-day on the ground tasks and responsibilities; and lived experience advocates were identified to understand how interventions can be more responsive to beneficiary and/or larger community needs. However, participants did not necessarily fall neatly into these siloed categories. For example, a lived experience advocate could have experience of leading an intervention; a leader could have experience of working as a project manager. Hence, an aggregate of their experiences while accounting for unique experiences and perspectives on implementation were considered and analysed.

#### 2.3.3 Sample size

The most common way of determining sample size for a qualitative study is through *saturation*. However, a low level of transparency regarding sample sizes and *how* saturation is assessed and achieved has been found in qualitative studies (35). The sample size in this study was guided by *information power* (36). This means that the more information the sample holds, the lower number of participants are needed. Information power in this study was guided by the specific study objectives (uncovering implementation experiences); characteristics of the sample (different perspectives on implementation); quality of the interview process (the depth of experiences uncovered); and the diversity of experiences within and across stakeholders and interventions.

Twenty-eight eligible participants were approached for the study, out of which 16 participants were interviewed (response rate—57.1%). A sample of 12–15 participants has been found to be adequate for a generic qualitative approach (29). There were seven participants from Australasia (two leaders, three implementors and two lived experience advocates). These participants represented three distinct complex interventions. There were five participants from Asia (three leaders, two implementors); who represented two distinct complex interventions. And four participants were recruited from different countries in Europe (four leaders). These participants were part of the same intervention but offered insights into its implementation within their individual countries. The sample included an equal number of men and women.

Out of the 12 eligible participants who were not interviewed, seven participants did not respond to our invitation (out of which one person had passed away), despite three reminders. Five participants declined to participate in the study: due to limited English speaking and comprehension skills, lack of availability, and not finding the study relevant to their current work. Most participants who did not respond or declined invitation were leaders (*n* = 11, and one lived experience advocate) and were from high income countries. A few of these individuals (*n* = 6) were from North America. And thus, interventions from North America were not included in the sample.

### 2.4 Data collection

Semi-structured interviews were conducted through an online video conferencing platform between August–November 2022. A written and verbal consent process was sought, after providing information about study procedures (Supplementary Material S1). The interview guide (Supplementary Material S2) consisted of open-ended questions on experiences involved in design, delivery and evaluating complex suicide prevention interventions, challenges, and the way forward. Separate interview guides were developed for the different types of stakeholders to understand their unique perspectives. The objective was to arrive at a holistic view regarding what happened on the ground, contributed by different vantage points.

All interviews were recorded using the platform and lasted for 60–75 min. The first draft of the transcripts was prepared using the interview platform. Transcripts were checked for accuracy against recordings and edited accordingly by the lead investigator (SK). Final transcripts were password protected and shared with respective participants for their inputs and edits. Data were deidentified and specific codes assigned to each participant. Caution was exercised in reporting and discussing any identifiable details (such as country/context, intervention name, professional background).

Most interviews (*n* = 13) were conducted by the lead investigator (SK) who has a master's degree in counselling, with experience and training in conducting qualitative research. The lead investigator had no prior relationship with these participants. She has had significant experience working on large scale community and institution-based implementation projects in low resource settings. As a result, she was familiar with the experiences shared by participants (especially implementors/project managers). These experience largely informed observations related to processes and challenges. The remaining interviews (*n* = 3) were conducted by a member of the research team (SM), with a PhD in Psychology and training and experience in qualitative research. Since these participants and interventions were known to the lead investigator (SK) the involvement of another interviewer was considered important to control for any potential bias.

Prior to the interview, interviewers (SK or SM) introduced themselves and the study objectives to the participants through an information sheet (Supplementary Material S1). This included the name of the interviewers, their position, qualifications, and role in the study. The sheet also contained information related to support services available in case of distress, tailored to the country context.

### 2.5 Data analysis

The data was analysed in two stages—the first stage involved multiple cycles of coding; and the second stage included an analysis of specific code categories using the NPT constructs. Hence, the analysis primarily followed a deductive approach. Deriving explanations of relevant phenomena (implementation mechanisms, outcomes, and context/environment in this study) through structured methods for data analysis using existing frameworks, models, and theories (37) has been found to be an important approach to integrating qualitative methods and implementation theory. A two-staged process to analysis helped—(a) navigate the vast volumes of data obtained from interviews; (b) ensure the analysis is responsive to the specific research questions in consideration.

The first stage involved multiple cycles of coding. First, line by line coding was conducted manually and a combination of codes were applied (38) to decipher the data. The second and third cycles of coding were conducted using NVivo (1.7.1, QSR International), which led to recognition of patterns, networks, and code categories. Analytic memos and a codebook were maintained throughout the process of conducting interviews and coding, to maintain consistency in codes and definitions used. Two investigators (SK and VR, PhD in Psychology) discussed and concurred on the emergent codes and themes. Broad code categories emerged from this process; for example—codes related to intervention approach, design, context. Multiple, individual yet interrelated codes were subsumed within these code categories. For this study, two specific code categories (named—*implementation process* and *context*) were chosen for further analysis. The *implementation process* parent code category comprised of all codes related to strategies and activities enacted in real world settings; ways of planning, ideation, developing protocols, executing plans into action—all action-oriented codes. The *context* parent code category included all codes related to multiple settings implicated in implementation—different levels and types of contexts.

**Figure 1:**
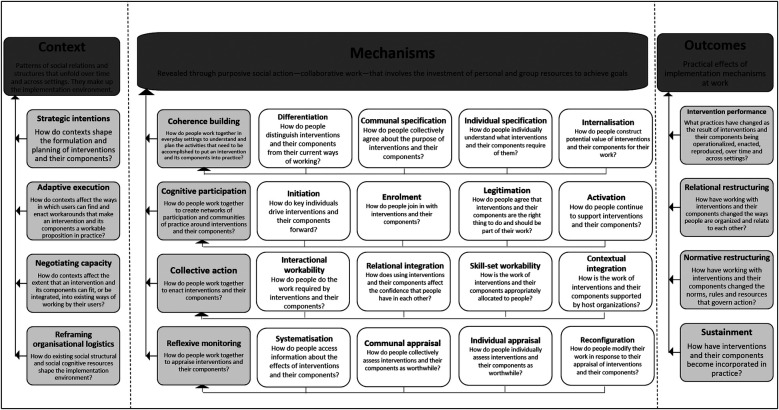
Summary of the coding frame: NPT domains, constructs, definitions and related questions.

In the second stage of analysis, data encoded within the selected code categories (*implementation process* and *context*) was then mapped onto NPT theoretical constructs. The NPT was considered appropriate for the study because it provides a set of tools to understand and explain the social processes through which new practices of thinking, enacting, and organising work are operationalized across diverse settings. NPT is popular because of its flexibility and applicability across a wide range of settings (39–41). In 2022, a translational coding manual for qualitative research was developed to clarify and simplify NPT to make it more easily applicable in research (27). The objective was also to facilitate transparent data analysis processes and reduce cognitive load involved in coding. The primary constructs are categorised into three domains—(a) *context*—defined as events in the system, unfolding over time in which the implementation work is done; (b) *mechanisms* (*coherence building, cognitive participation, collective action, reflective monitoring*)—that motivate and shape the work people do; and (c) *outcomes*—effects of implementation work within a context (27). There are 12 primary constructs subsumed within these three domains. The secondary constructs (*n* = 16) further elaborate on the four primary *mechanisms* outlined in the theory (see Figure 1).

The objective of the second stage of analysis was to use this body of constructs (and the accompanying guide questions) to examine *mechanisms* that motivate and shape implementation processes, the *outcomes* of these processes and the *contexts* in which their users/stakeholders make them workable and integrate them into practice. For the *context* and *outcomes* domains, excel sheets with their respective primary constructs, associated definitions, and guiding questions, were created. With respect to the mechanisms domain, separate sheets were created for each of the four primary constructs: *coherence building*, *cognitive participation*, *collective action*, and *reflective monitoring*. In each of these sheets, their respective secondary constructs were enlisted along with their definitions and guiding questions. The mechanisms domain was mapped first. Each statement coded under the previously identified code category on *implementation process* was mapped onto the guiding questions under secondary constructs. An aggregate of responses to the questions under secondary constructs, led to an answer for the main question under a primary construct. Therefore, mapping proceeded from more specific to general constructs as indicated in Figure 1. For example, the aggregate of responses (statements coded) to questions under the secondary constructs—*differentiation, communal specification, individual specification, internalisation* provide an answer to the main question asked under primary construct of *coherence building*—*How do people work together in everyday settings to understand and plan the activities that need to be accomplished to put an intervention and its components into practice?* For the context and outcomes domain, data was mapped onto primary constructs in a similar manner. These domains did not have secondary constructs. Approximately 10% of these codes were checked by another team member (KK). At the end of the process, themes within responses (statements mapped under constructs) to these questions were identified. These themes directly address our research questions shedding light on how intervention programs were implemented and the factors contributing to their *successful* delivery. A more detailed discussion of the challenges (42) and barriers (43) is presented elsewhere.

## 3 Results

An overview of the intervention characteristics, context and approach to intervention delivery has been summarised in Table 1. While most interventions were implemented in high-income contexts (HIC), one intervention was implemented within a low and middle income (LMIC) context. Interventions were population based and implemented across different settings such as healthcare, school, community, and service industry; and were delivered by multiple participating stakeholders.

**Table 1:** Overview of context, intervention characteristics and approach to intervention delivery.

Intervention context and characteristics	Intervention 1	Intervention 2	Intervention 3	Intervention 4	Intervention 5	Intervention 6
Country setting	Australasia	Australasia	Australasia	Asia	Asia	Europe
	High income settings	High income settings	High income settings	Low and middle income settings	High income settings	High income settings
Intervention components	Nine components including a school program, training programs for stakeholders and healthcare staff, community awareness programs	Multiple components including initiatives for high-risk groups, awareness campaigns, training programs for stakeholders, school program, means restriction campaign	Five components including general and specialised training at different levels and ongoing support for bereavement	Three components including a school program, community means restriction program, training program for healthcare staff	Four components including a community awareness program, mental health consultations, financial support, and other health promotion activities	Four components including training programs for stakeholders, facilitation of referrals, media campaign
Intervention levels/settings	Population based: Implemented at universal, selective and indicated levels of prevention	Population based: Implemented at universal, selective and indicated levels of prevention	Service industry setting: Implemented at universal, selective and indicated levels of prevention	Population based: Implemented at universal, selective and indicated levels of prevention	Population based: Implemented at universal, selective and indicated levels of prevention	Population based: Implemented at universal, selective and indicated levels of prevention
Target population	All age groups, different sub-populations	All age groups, different sub-populations	Diverse age groups—engaged in public and private sector service industry	All age groups, different sub-populations	Aimed at the general population but primary target was middle-aged and aged population	All age groups, different sub-populations
Intervention delivered by	Program staff, community members, organisations	Program staff, community members, healthcare professionals	Program staff, trained field officers	Program staff, community health workers, community members	Program staff, community members and healthcare professionals	Program staff, community members and healthcare professionals
Institutional support	Yes, implementing institution/organisation responsible for recruitment, capacity building and liaising with community stakeholders to offer support for delivery of intervention and its components.	Yes, a central implementing body involved. Partnerships with support provided to local implementing organisations (Primary health networks, non-government, and community organisations etc.) to implement the intervention and its components. Implementing organisations participated in decision making about the conduct of the trial.	Yes, charity established by the people of a specific service industry. Intervention designed by and delivered for the people of the service industry.	Yes, implementing institution/organisation responsible for recruitment, capacity building and liaising with community stakeholders to offer support for delivery of intervention and its components.	Yes, implementing institution/organisation—responsible for capacity building and support for delivery of the intervention, conducting trial evaluation.	Yes, implementing institution/organisation—responsible for capacity building and support for delivery of the intervention, conducting trial evaluation across different countries in Europe.
Approach to intervention delivery	The design of the intervention was pre-determined by the implementing institution. Program staff were hired by the implementing institution to work in collaboration with community stakeholders across settings to implement the intervention as per protocol. Fidelity to the intervention design was of utmost importance because this included an effectiveness trial.	The implementing institution worked directly with community stakeholders across settings to implement the intervention. Important community stakeholders were made responsible for delivery of intervention activities, with support from the institution. Stakeholders had a choice between two evidence based complex (multilevel and multicomponent) intervention designs. The focus was on ensuring reach, adoption, quality more than fidelity to intervention design.	The intervention was developed and delivered by people of the industry, with other community partners offering support for the capacity building components of the program. The focus was on ensuring reach and adoption of the training programs, more than fidelity. However, the adopting organisations needed to implement the intervention as a whole and not the individual constituent parts.	The design of the intervention was pre-determined by the implementing institution. Different levels of program staff were hired by the implementing institution to work in collaboration with community stakeholders across settings to implement the intervention as per protocol. Fidelity to the intervention design was of utmost importance because this was an effectiveness trial.	The local government worked with an educational institution to develop a design for promotion of well-being within the local community. The intervention fit well within the government's policy to reduce suicide rates within their jurisdiction. The implementing institution worked directly with community stakeholders to deliver intervention activities.	The design of the intervention was pre-determined and was evidence based. Hence, the intervention package was scaled up across different countries. The central implementing institution insisted on ensuring fidelity to the intervention package, with adaptations to the country setting. In each country, staff were hired to anchor intervention activities in collaboration with community stakeholders across intervention settings.

Across all interventions, an implementing organisation (which typically received funding for program activities) anchored the design, delivery, and evaluation of the intervention. Some interventions adopted a tiered approach, wherein the implementing institution recruited local staff tasked with liaising and collaborating with stakeholders in various settings to ensure the delivery of intervention activities according to a pre-established protocol. In other instances, the implementing institution worked directly with community stakeholders and provided support for delivery of intervention activities.

Following are inferences and insights drawn from participant reflections regarding the influences on complex suicide prevention interventions across the three domains of the NPT (Figure 1). These three domains address our main research questions.

### 3.1 How does the context influence complex suicide prevention interventions and their implementation environment?

For this study, contexts were understood as dynamic networks of social relationships and structures that make up the implementation environment. The dynamic interaction between the practices and resources embedded within a context and the expressions of agency of the stakeholders involved in implementation is reflected in the findings. The focus was not on enlisting determinants but understanding the nature of influence and the role stakeholders play in negotiating these influences. This includes influences on formulation and planning of interventions (primary construct—strategic intentions), workarounds and adaptations (adaptive execution), integration into existing practices (negotiating capacity), as well as social-structural and social-cognitive resources that make up the implementation environment (reframing organisational logistics) (see Table 2).

**Table 2:** Contextual influences on the implementation environment.

Primary constructs— context	The dynamic elements of the context	*How* do these influences impact the intervention and its components?	Illustrative quote
Construct 1: Strategic intentions How do contexts shape the formulation and planning of interventions and their components?	• Context needs—to address structural policy issues, sub-population specific needs, priority settings, gaps in services/appropriate programs, lack of local evidence; emerging needs—during the course of the intervention • Local community practices (such as crop sowing and pesticide use patterns) • Strengths, protective factors and opportunities—existing within communities or the intervention setting which could enhance the intervention • How suicide is perceived and understood	• Choice of programs (components, strategies) • The approach towards program implementation (top down, bottom up) • Evaluation plans	“OK, look, if everyone's saying reducing access to means works, let's see if we can in [country name]…Reducing access to means is really about reducing access to pesticides here. So can we reduce access to pesticides? Oh, now many people would say that that requires policy interventions” (Leader 03)
Construct 2: Adaptive execution How do contexts affect the ways in which users can find and enact workarounds that make an intervention and its components a workable proposition in practice?	• *Adapting intervention characteristics:* Enacting adaptations to the intervention, such as modifying the scope of the intervention, the focus, nature of intervention materials informed, efforts such as ongoing training and ensuring continuity in staff • *Adapting context related characteristics:* Choosing sites where there are existing relationships to leverage, even prior to introducing the intervention, understanding what is acceptable/not acceptable for people, understanding the healthcare system, socio-political-economic environment, tiered communication strategies for different stakeholders and their needs, shifting organisational culture, ways of doing things	• Enhancing acceptability of interventions • Enhancing feasibility of implementing interventions within resource constraints • Enhancing responsiveness to context needs • Creating accommodations, facilitating adoption of interventions.	“One of the things that we did when selecting the sites was…we kind of made it a requirement that they had to have preexisting relationships. What we needed to see was evidence that they already had at least some pre-existing relationships with those other organizations who are working in the sector” (Leader 05).
Construct 3: Negotiating capacity How do contexts affect the extent that an intervention and its components can fit, or be integrated, into existing ways of working by their users?	• Leveraging pre existing relationships, collaborations and networks. • Negotiating with local policy makers • Engaging local expertise • Facilitating an understanding regarding the need for the intervention. • Aligning the goals of the intervention with the most salient problems faced by communities—integrating the intervention into existing programs/systems.	• Enhancing acceptability of interventions • Facilitating transitions to new ways of functioning, new practices	“The [type of] industry was a bit unique in that the industry had an…employee assistance program…Any worker who are member of one of the unions had access to 16 h worth of free counselling…for them and their family. They could access it by just calling up and giving their union number. So we actually had the clinical support team and so we knew that we didn't have to do that [as part of the intervention program]. We just had to get people there because no one was using it…” (Lived experience advocate, 02)
Construct 4: Reframing organisational logics How do existing social structural and social cognitive resources shape the implementation environment?	*Macro:* A. Legal/policy: Legality around suicidal behaviour; nature of funding that is considered acceptable (e.g., surveillance on foreign funding); changing rules of operation; access to data on suicides. B. Political/bureaucratic environment: Day to day operations; existing power structures; changes in governance. C. Socio-cultural structures: Inherent social structures and norms/rules of engagement; how the intervention is conceived by the beneficiaries (relative value, merit and worth); how suicide and mental health are perceived and defined; beliefs/perceptions regarding necessary actions to be undertaken to address suicides; perceptions about whose responsibility it is to prevent suicides.	A. Reporting of suicidal behaviour; quality of data on suicides. B. Nature of stakeholder relationships and involvement/participation in delivery of programs; sustainability of programs. C. Nature of engagement in programs; acceptability of interventions; messaging around what the intervention entails; implementation strategies (problem solving and mitigation strategies); nature of relationships; access into communities; engagement of lived experience	“…In any community work…you work with a set of officials/bureaucrats, and they can be helpful and you are almost there. And then suddenly they get shifted and then the other person doesn't want to continue with whatever you have done, for example, to get the permission to train community health workers…So, we have to go higher up” (Leader 06) “People don't really talk about mental health and suicide…when asked, they don't openly discuss these topics. And that is why this kind of community engagement is necessary. Only through such community engagement can we begin to understand what people are experiencing and going through in their daily lives…So…to run an intervention like this, it is important to be on the field with people…to really be able to understand what they are going through” (Implementor 01).
	*Meso:* A. Research and academic environment: understanding around etiology of suicide; understanding regarding important measures to prevent suicides. B. Organisational environment: infrastructure, capacity, time, and funding. C. Intervention related: complexity and design	A. Defining priority areas for intervention; who is involved in suicide prevention efforts. B. *Infrastructure and funding* impact: geographical access to sites; amount of time for intervention activities; access to services within communities; nature of activities undertaken; recruiting and supporting capacity; quality of dissemination; sustainability of activities; nature of evaluation; collaborations and networks. *Time* impacts quality of engagement of stakeholders in the program; quality of delivery. *Capacity* impacts: quality of delivery; scale; sustainability; quality of linkages, networks, relationships; guidance and direction regarding how activities should be implemented.	
	*Individual:* • Stakeholder engagement (individual and collective), willingness to engage, beliefs about the intervention and what it entails.	Acceptability; quality of delivery	

Participants discussed their *strategic intentions* (primary construct 1) which is their (individual and collective) initiation of new processes/practices with the *intention* of creating a new outcome. Reflections related to how contextual considerations shaped initial intentions related to intervention design and planning for delivery, were shared. Insights related to—*what about the context impacts the intervention*, and *which features of the intervention are impacted*, emerged. Perceived gaps such as addressing the needs of specific population sub-groups and priority settings (adolescents, farming and rural communities, health workers, and other marginalised groups); addressing policy related issues in the investment towards and conduct of suicide related work; gaps in existing services; socio-cultural determinants of suicide, as well as the need to develop a more culturally responsive intervention, were a few examples shared. In many settings, stakeholders (especially implementing organisations) tended to operate on the assumption that the choice of interventions is largely based on internally recognised evidence base. Furthermore, other contextual characteristics, such as local community practices (e.g., crop sowing and pesticide usage patterns); leveraging community strengths and protective factors (e.g., support and connectedness); and community perceptions of suicide, were noted to impact the selection of intervention programs (including components and strategies), the choice of settings (community, school, healthcare, etc.); the approach to delivery and adoption (top-down, bottom-up, participatory, etc.), and evaluation plans.

Contexts were negotiated and shaped by the actions of the participant stakeholders as they worked through the relational and normative environment. Participants described the influence contexts have on their *adaptive execution* (primary construct 2)—deliberate efforts to make interventions work. Workarounds related to intervention characteristics included adapting the scope of interventions (number of components and activities to be implemented) as well as the focus of intervention activities (limiting objectives, whether focus on primary prevention or strengthening tertiary systems). Other efforts at adapting interventions to fit the context included tailoring intervention materials; ongoing training for staff to understand what the intervention entails; and adopting a tiered communication approach with different stakeholder groups. Participants also chose to negotiate with some aspects of the intervention environment itself—specific intervention sites with pre-established relationships were chosen to facilitate adoption of the intervention. Conducting a needs assessment; negotiating with local authorities; inviting local expertise to understand adaptation needs; and facilitating conversations around the need for such an intervention were other ways of creating accommodations for the intervention.

Contexts also influence the *negotiating capacity* (primary construct 3) or the extent to which an intervention can be integrated into existing ways of working and thinking. Participant narratives were reflective of efforts at leveraging pre-existing relationships and collaborations between stakeholders involved in implementation, using local expertise and resources to accommodate the intervention. Aligning the goals of the intervention with the most prominent issues concerning a community of beneficiaries was another way. The most prominent strategy for ensuring integration of an intervention was piggybacking on an existing system or program, which was an important way of ensuring complementarity of interventions with existing programs and systems.

While contexts influence stakeholders' capabilities to enact workarounds, there are inherent dynamic elements within the context that fundamentally shape the intervention environment (*reframing organisational logics*—primary construct 4). An understanding of many social-structural and social-cognitive resources across levels of contexts emerged through participant narratives. At the macro level (broader population/system level), factors such as the policy and regulatory environment impacting (de)criminalisation of suicides, surveillance systems and economic policies impacting funding and priority areas of policy engagement were highlighted. Participants also shared how bureaucracy at this level impacts operations, sustainability of programs as well as mandates around who participates in the delivery of interventions. At the meso level (community, organisation), stakeholder characteristics, existing networks and linkages, academic environment, resources, were found to impact a range of decisions such as choosing programs for implementation, nature of activities and collaborations, quality and scale of implementation, sustainability of programs. At an individual level (stakeholders, groups), engagement and investment in the intervention and its goals, collective readiness, and shared commitment to addressing suicides was reported to be important determinants of intervention success (or failure).

### 3.2 How do agents (stakeholders involved) make sense of, commit to, execute, and evaluate complex suicide prevention interventions?

We aimed to uncover the *mechanisms* underlying the work (investment of personal and group resources to achieve goals) involved in implementing complex suicide prevention interventions. This section primarily examines mechanisms of *success* and deliberate strategies employed to support them.

Within NPT, mechanisms are understood to motivate and shape the work that people do when they participate in implementation processes (see Figure 1). The focus was on *how* the work gets done through shared meanings and a sense of competency (primary construct 5—*coherence building*); engagement and enrolment into the practice (primary construct 6—*cognitive participation*); organising and enacting a practice (primary construct 7—*collective action*); and formal and informal evaluation of implementation processes (primary construct 8—*reflexive monitoring*). Most importantly, the investment of *meaning*, *commitment*, *effort*, and *comprehension* respectively, was understood to drive practice. The influence of these mechanisms (represented by the four primary constructs and 16 secondary constructs) in energising implementation processes is discussed below and summarised with quotes from the study participants in Table 3. Layers of complexity emerged in the data—investments made by participants interviewed in the study could be observed across the implementing organisation and community level; and across pre-implementation and implementation/roll out phases. Although several definitions of the term *community* exist, in this study—the term was used to refer to all stakeholders and settings which were understood to be the target of change.

**Table 3:** Overview of mechanisms involved in implementing complex suicide prevention interventions.

Primary constructs- mechanisms	Secondary constructs	*How* do these mechanisms impact the way interventions and their components are put into practice?	Illustrative quotes
Construct 5: Coherence building (meaning making—what is the work?) How do people work together in everyday settings to understand and plan the activities that need to be accomplished to put an intervention and its components into practice?	Differentiation: How do people distinguish interventions and their components from their current ways of working?	• At the implementing organisation level: people distinguish through organised training and onboarding activities. • At the community level: information about the intervention and its components by the implementing organisation. Dissemination channels and the content of the message can be different for varied stakeholder groups. Training and capacity building on the intervention also helps people understand how it is different from what they are currently doing.	“And I would say Tier 2 or tier T3 level stuff, so including say field based staff or someone who does the data collection or someone who is a local data manager…they also undergo an onboarding process…It's just sometimes we just have to tone it down because the educational qualification and the background does play a major role when we are giving them information about the intervention. So if it is a research associate level entry, then we have slightly different content…” (Implementor 01).
	Communal specification How do people collectively agree about the purpose of interventions and their components?	*Pre-implementation phase:* • At the implementing organisation level: agreeing on an implementation plan and the details of what needs to be done, timeframe etc; conducting a needs and gaps analysis, identifying priority areas, choosing programs, carving out an intervention identity, defining the limits of the intervention helps in creating a common understanding. • At this stage, the community can be invited to contribute to any of the above. Apart from this, any efforts toward mobilising the community towards a common cause can help in creating a common understanding regarding what is needed to be done.	“What was that going to look like? Deciding which bits would be…Evidence and evaluation pieces, which bits we already knew from national evidence worked, and we wanted to implement and fund straight away. So then it was quickly identifying what would be commissioned, and then it was performance managing the commissioned activity.” (Implementor 04).
		*Implementation phase*: • Several activities initiated by the implementing organisation (and the participating community) can help create a common understanding about the collective purpose. This includes: announcing the intervention, training and capacity building activities for the community, mobilising the community regarding common responsibilities, disseminating a common message, mobilising and lobbying with the local government can help develop a common message. • At the implementing organisation level: setting up systems, training and capacity of staff in protocols, regular meetings to trouble shoot problems, keeping different levels within an organisation updated.	“And so that you prepare for opening ceremony opening ceremony is something. In the City Hall, in a public place…I always go out into community…you make a big opening ceremony with music and art…and some talks related to why the intervention is important…and the next day you should start with your public relation campaign with the trainings with your four level intervention” (Leader 01).
	Individual specification How do people individually understand what interventions and their components require of them?	*Pre-implementation phase:* Within the implementing organisation—formulating implementation plans, directions from the leadership as well as efforts in defining roles and role boundaries helps people understand what is required of them.	“Yeah. So, the implementation plan was developed by the implementation guys to have a plan for how to roll out activities…They were developed in collaboration with the four sites” (Leader 05)
		*Implementation phase:* • Different ways in which members of the community and the implementing organisation become familiarised with what is expected of them: helping them understand similarities between the intervention and what they do; training and capacity building in implementing the intervention and offering support; and through dissemination and information sharing. • Within an implementing organisation: several systems and structures in the form of monitoring and governing bodies, protocols; as well as processes such as training and capacity building, monitoring and supervision, collective problem-solving help individuals understand what is required of them.	“In the program, specialist training is offered to the public health staff…so that they are aware of the local health promotion plan in each town…which forms an important part of the intervention…” (Leader 08)
	Internalisation How do people construct potential value of interventions and their components for their work?	*Pre-implementation phase:* • At the community level, building a community buy in for the program: by listening to their needs and wants, and using specific strategies for communicating how the program fits, can help build its relative value and merit. • At the implementing organisation level, conducting a needs analysis, understanding the context, developing a program identity as well as pitching the program to external stakeholders can help people build an understanding of the relative worth of the program.	“The second issue was when you look at the industry from an employer point of view…It is hyper competitive, it's really every job is one and low margin, tight time frames. So for employers saying we're not gonna take on another program like we already got workplace health and safety like why on Earth would we buy into another risk? We talked to unions and employers…there's absolutely no trust between the…so we had to listen to their issues to understand whats going on” (Lived experience advocate 02).
		*Implementation phase:* • At the community level, several activities like inviting stakeholder opinion, building community responsibility and ownership, training and capacity building, lobbying with the local government, and specific communication strategies can help in understanding the potential importance of the intervention. • At the organisational level, several structures and activities like training, mentoring and supervision, monitoring meetings, systematic evaluations of progress and impact can help understand the potential value of the intervention.	“Partly engagement, but was trying to keep…Even when you had these meetings, because they might only be 15 min of the meeting is relevant to that one person. So just trying to work out the information flow so that people had the information they needed to be able to make informed decisions. And we needed them to make decisions so that they could have ownership in what we were doing.” (Implementor 03)
Construct 6: Cognitive participation (commitment—who does the work?) How do people work together to create networks of participation and communities of practice around interventions and their components?	Initiation How do key individuals drive interventions and their components forward?	*Pre implementation:* There are many activities key individuals within implementing organisations engage in to drive the intervention: looking for funding, conducting a needs analysis of local needs; seeking permissions from local governing bodies; defining limits and scope of the intervention; developing a clear implementation plan; defining what needs to be adapted vs. standardised. An important driver at this stage of implementation is developing a buy in from the community for the intervention through community consultations and feedback.	“And we started with those norms and we then went out to industry and say what you experience, what are you seeing? What are you feeling? What's happening?” (Lived experience advocate 02).
		*Implementation:* • At the organisation level, activities such as developing protocols, guides and systems; training and onboarding of staff; on the ground activities such as planning, coordinating logistics, liaising with different levels of the organisation; and troubleshooting, monitoring and supervision activities help drive the intervention and its components. • At the community level, activities such as utilising existing structures and systems; building capacity of stakeholders; building ownership by inviting feedback and participation; disseminating information about the intervention and its activities through channels; and lobbying with local policymakers helps drive the intervention and its components	“So basically we have a very structured protocol because [funding organisation] mandates that. So for every profile given in the protocol, we follow a set of guidelines. So for example, when we were onboarded, the important thing was to just get acquainted with the protocols, various procedures because they have different components…” (Implementor 01).
	Enrolment How do people join in with interventions and their components?	*Pre implementation:* • At the organisational level, activities such as developing systems and structures, can help staff enrol in the intervention and its components. • At the community level, activities such as conducting a needs analysis, systematically building a buy in—a consultation with stakeholders about what needs to be done, stakeholder specific communications strategy can help them enrol in the intervention and the components.	“But you know they are very suspicious of people coming in from…the cities and people coming in from the government and not really being genuinely connected or caring about their community, especially in rural areas, so people have to be on the ground, going out, visiting…very time consuming and also requiring your resources, quite practical resources around transport and…You know, satellite phones and…Accommodation and all of that.” (Leader 02).
		*Implementation:* • Several activities like announcing the intervention; training and capacity building for community members; building ownership; utilising existing structures and systems; having a planned dissemination strategy; onboarding and partnering with local governments can help community stakeholders participate in the intervention. • At the organisation level, this involves activities such as regular meetings, keeping updated regarding field activities, monitoring progress, mentoring and supervision	“You know, we had to work with the community…and in the initial stages itself we told them the community has to give a space, so it…they become a partner in that. It's not that we were…whereas…in the WHO site…we rented the place…but here we wanted a partnership so that it will be sustainable. So we said the community has to give us the space and we will provide the [intervention materials].” (Leader 06)
	Legitimation How do people agree that interventions and their components are the right thing to do and should be part of their work?	*Pre implementation:* • Literature on effective interventions as well as pragmatic reasons can guide decisions on what to do and the course of action at the organisation level. • At the community level, activities such as collaborative gaps analysis, communication strategies to build a buy in can help in onboarding stakeholders	“And then we looked around and we see training of GPs and there were studies. Access to lethal means and the general public and stigma and all the different obstacles to treatments…interventions. And so we came with a bunch of interventions and I tried to get a certain order in it by defining 4 levels. This is a little bit arbitrary but…6 levels is too much for the human brain; 2 is not enough. So we started with more or less four and I think this was a good idea.” (Leader 01)
		*Implementation:* • Processes such as lobbying with and seeking an official mandate from local governments; utilising existing resources, systems and structures; training and capacity building of stakeholders; systematic dissemination of intervention related information; building ownership and responsibility for the intervention are ways to establish its legitimacy at the community level. • At the organisation level, activities such as ensuring a rigorous oversight, review and monitoring of quality and progress helps establish legitimacy.	“Our study is carried out with the cooperation of the local government…So the officer of the local government would like to do a policy package of the suicide prevention because as you know, they make policies but don't know how to conduct them…But due to the possibility of reducing suicide, they asked me to do such kind of intervention in our area…”(Leader 08)
	Activation How do people continue to support interventions and their components?	• At the community level, activities such as onboarding and ensuring support from local govts; continuous training and capacity building of community members; building ownership and responsibility for the intervention; utilising existing systems; communicating a consistent message about the intervention and its progress are important ways through which people continue to support interventions and their components. • At an organisational level, sustained funding, developing systems and structures for daily functioning; adopting an action framework and creating workgroups; liaising with different levels within an organisation; training and capacity building of staff; regular meetings to troubleshoot problems; examining implementation to understand what works and what does not—helps people to continue to support the intervention.	“So…They knew me and they said no, that we…we want…Uh, because our plans were not like that. Our plans were to train one GP or two GPs and then they would train. So it was train the trainer…and then they would train the cohort. so that the training continues” (Leader 07)
Construct 7: Collective action (effort—how does the work get done?) How do people work together to enact interventions and their components?	Interactional workability How do people do the work required by interventions and their components?	*Pre implementation:* Seeking permissions from authorities to implement, establishing partnerships, conducting a thorough needs analysis, developing an implementation plan—understanding what needs to be standardised vs. adapted and defining role boundaries can be seen as doing the work required by the intervention	“So for example, we got the state government to become a partner when we were applying for the grant. So that meant that we had done a lot of the conversations at the state level even…even before when we applied for the grant. They gave us a letter saying they were committed it to it and they were partners on it, and they would want to work with us.” (Leader 03)
		*Implementation:* • At the organisation level: several day to day activities involving planning, coordination, managing logistics; setting up systems and structures to carry out tasks; designing protocols and intervention guides; procedures for check ins and problem solving—such as meetings; procedures for reflexive monitoring through formal and informal processes keep the intervention going. • At the community level-onboarding local authorities as partners; embedding the implementation in existing structures and systems; building ownership and responsibility for the work within stakeholders; training and capacity building of stakeholders in the intervention and protocols; disseminating information about the intervention, its progress are activities that keep the intervention going.	“Trying to do some engagement but also management of stakeholders, because we're trying to give them information while also the information was changing and evolving but also working with the [implementing organisation] to help…Actually…Either choose the interventions…or work towards different guides about the interventions for the trial sites. Some of the other trial sites helped them develop those as well. So that was part of it.” (Implementor 05)
	Relational integration How does using interventions and their components affect the confidence that people have in each other?	Understanding role boundaries; building ownership through capacity building and collective philosophy; using different channels for communication; communities of practice; reflecting on the intervention process; supervision spaces.	“I think the other part…you know communities of practice are a bit of a fad at the moment. Everyone loves a good community of practice. But I think they do have a…I…I think…I think they have a role, if not a pivotal role in complex interventions… hearing the perspectives from the other site coordinators was super useful in us working out what we needed to do ourselves, exactly, and there was…Cross fertilization of ideas…we were really engaged in that problem solving discussion about, well, how would we do it? How could we do it?” (Implementor 03)
	Skill set workability How is the work of interventions and their components appropriately allocated to people?	• At the organisational level, developing a roll out plan; setting priorities and role boundaries; creation of specific governance and workgroups; day to day management involving logistics, coordination and planning; establishing protocols and guides; regular meetings to keep track of activities. • At the community level, building ownership through specific activities and developing stakeholder specific communication strategy.	“I now have to go into each hamlet within a village and conduct meetings with community members regarding the interventions. For instance, for [intervention component], we tell them about the facility and give them information regarding its benefits and how they can use the facility within their own village. So these kind of tasks are conducted with different communities within a village. There is no fixed task as such. Our work is to mobilise the community to become a part of the interventions. My work changes each day.” (Implementor 02).
	Contextual integration How is the work of interventions and their components supported by host organizations?	Organisations can support the intervention and its activities in many ways: initial consultations with stakeholders; facilitating permissions; expert advice on governance; funding for activities; building ownership; training and capacity building; embedding the program within existing systems; lobbying with local governments.	“And looking at how we embedded, supported the activity across our [implementing organisation], so embedding it across all of our portfolios within the [implementing organisation]. So our data, our finance, our mental health team, our commissioning team, our practice support our GP network, our practice support team and our population.” (Implementor 04)
Construct 8: Reflexive monitoring (comprehension—how is the work understood?) How do people work together to appraise interventions and their components?	Systematisation How do people access information about the effects of interventions and their components?	*Pre implementation:* Activities such as stakeholder consultations and reports, developing stakeholder specific communications strategy and establishing systems for data collection can ensure people can access information about the effects of the intervention	“Made data reports or just consultation reports with key recommendations that we'd heard. We then dived down further into producing those consultation reports for each of those separate communities within the trial and so that each community could see what their community was saying and what they were identifying as the needs. And then if they agreed to that, we would then now start to work towards those identified needs or the recommendations from each of the…the regions.” (Implementor 04)
		*Implementation:* • At the organisational level, there are different mechanisms through which staff can access information about the intervention and its effects—developing a tracking and data collection system; asking questions about the implementation regularly to understand how and why; Developing feedback mechanisms within the organization and the community; documenting activities on the ground to understand what happened; developing monitoring and oversight mechanisms; conducting a systematic evaluation of the factors influencing implementation as well as the overall impact; regular meetings for troubleshooting problems; through communities of practice for collective and iterative problem solving; having governing and oversight bodies to look into the quality of implementation. • At the community level, developing different communication channels, planning for dissemination to ensure the community stakeholders are kept in the loop and informed about the implementation are some of the ways for them to know about the intervention and its effects.	“That's how the nature is, because most of the time we are concerned about the efficacy, but we have to remind ourselves that this is more of an acceptability as well as effectiveness trial. So you sometime have to just document the process, try to come up with something that doesn't alter the entire design, but you also have to be more focused about how you can make this more…I would say user friendly.” (Implementor 01)
	Communal appraisal How do people collectively assess interventions and their components as worthwhile?	*Pre implementation:* Community consultations can be ways of collectively assessing if the intervention is worthwhile.	“When we first engaged with the community, they were really keen to tell us what wasn't working in their communities and we were keen to hear that as well. But we had to work really hard to change that narrative into what solutions did they think that their communities had” (Implementor 04)
		*Implementation:* • At the organisation level, there are different ways in which people collectively assess whether the intervention is worthwhile—developing protocols for procedures; developing systems for tracking data; regularly monitoring quality; documenting what happened on the ground; asking questions about the nature of implementation; conducting regular meetings to troubleshoot problems; conducting a systematic evaluation of the program; having governance and oversight bodies; through communities of practice for collective and iterative prob solving. • At the community level, this involves informing the community about the intervention and making decisions in consultation with the community	“Very messy. Yeah. And look, we…we have gone some way towards capturing some of that implementation related data… But I have to say I think that there's still gaps in in that. Umm. And where actually. So one of our team is writing a…A paper…Probably be higher level around—What happened? The sites probably did document a lot. A lot of this stuff. Umm. Couple sites in particular…did a lot of work around documenting this” (Leader 05)
	Individual appraisal How do people individually assess interventions and their components as worthwhile?	By sharing and receiving information about the intervention progress and effects; receiving feedback from higher authorities; through conversations about what worked and what did not; in the context of mentoring and supervision; documenting activities on the ground; through their own reflections in communities of practice; in the context of meetings; and through feedback from monitoring of quality and progress	“And so you need…You need sort of enough…I don't know if enough is the right word…enough capacity for reflective practice. Umm to be able to work out what are these problems? Is that project related? Is that related? Is that them related? Is it intervention related like…what are the factors that are causing a problem? Yeah, I think they are the main things.” (Implementor 03)
	Reconfiguration How do people modify their work in response to their appraisal of interventions and their components?	Enacting changes in the implementation plan, changing the approach to the delivery of the intervention; reassessing the scope of the intervention; using feedback to solve problems; gathering support from local authorities; using this support for conflict resolution; managing and relaying protocol deviations within levels of the organisation.	“So let's say for an example, if you have to collect the data that is supposed to be done in a group setting, but because of the COVID related thing, we couldn't conduct a group level data collection because the schools were closed. So then the question was how can we do that? So in those particular scenarios, we came up with a slight modification in the plan like can we do door to door data collection.” (Implementor 01).

#### 3.2.1 Coherence building

Participants shared their experiences and ideas about building a shared sense of understanding regarding the meaning and uses of the intervention and what it entails (primary construct 5—*coherence building*). Specific organised and systematised features of the context (both individual and collective) such as shared standards or norms; skills, knowledge and expertise to perform tasks; as well as opportunities for demonstrating these skills contributed to this shared meaning. An important way stakeholders invested meaning in an intervention was by *differentiating* (secondary construct 1) it from existing ways of working. At the organisational level, onboarding and training activities for staff helped understand the distinct, characteristic features of interventions. At the community level, dissemination of information about the intervention and training and capacity building support by the implementing organisation for community stakeholders contributed to a sense of differentiation. Meaning was also invested by collectively agreeing about what the intervention entailed (*communal specification*—secondary construct 2). This varied across implementation phases and social-ecological levels. In the pre-implementation phase, organisations developed implementation plans, defined the scope and limits of the intervention, and conducted needs analysis to arrive at a common understanding of *on the ground* issues and what needed to be done. A few interventions invited collaborative participation of community stakeholders in these processes. This contributed to a common sense of understanding about the intervention and its components. This differed in the implementation phase, which entailed an ongoing investment in collective meaning making. While developing protocols, staff training and regular meetings to troubleshoot problems were important ways of building coherence at the organisational level; other activities initiated by the implementing organisation such as mobilising the community regarding common responsibilities helped at the community level.

Apart from collective meaning making, participants also reflected on how they understood (as individuals) what was expected of them (*individual specification*—secondary construct 3). In the pre implementation phase, implementation plans, definitions around role boundaries, and directions from the leadership helped in understanding what was expected out of everyone at the organisational level. In the implementation phase, drawing similarities between what community stakeholders did and the intervention activities helped them understand what was expected of them. This was also supplemented with dissemination of information and capacity building of community stakeholders to understand their roles and responsibilities. At the organisation level, protocols, procedures and meetings continued to help staff understand their roles and responsibilities. Participants also constructed the potential value of the intervention and its components (*internalisation*—secondary construct 4) through developing a program identity, pitching the benefits of the program at all levels within the intervention environment and using strategies to communicate how the program fits into the context, in the pre-implementation phase. However, this was different in the implementation phase, as it involved activities such as inviting stakeholder opinion on the delivery of the intervention, lobbying with local governments and building community responsibility and ownership of the intervention. This helped build the value of the intervention at the community level. Within the implementing organisation, mentoring and supervision, check-ins through meetings, and systematic evaluations of progress helped in understanding the potential value of the intervention.

#### 3.2.2 Cognitive participation

Participant experiences were reflective of their *cognitive participation* (primary construct 6) in the intervention, or the processes involved in preparing them for working together effectively and their real and symbolic involvement in the intervention. In the pre implementation phase, participants engaged in processes such as seeking funding, conducting a needs analysis, deciding what needs to be adapted, at the organisation level. Building a community buy in from the community through consultations and feedback helped in the process of *initiation* (secondary construct 5) or driving the intervention forward. In the implementation phase, initiation involved activities related to developing and following protocols, onboarding, and training, managing logistics, at the organisation level. At the community level, the focus was on utilising existing systems and resources, building capacity of stakeholders and lobbying with local policymakers to drive the intervention and its components forward. Furthermore, there were many ways in which stakeholders worked together to organise themselves to participate in the intervention (*enrolment*—secondary construct 6). This involved processes such as developing systems and structures and developing stakeholder-specific communications strategy prior to implementation. Other activities such as formal announcements regarding the intervention, onboarding and partnering with local stakeholders, and monitoring progress contributed to enrolment of stakeholders in the intervention and its components.

There seemed to be a conscious process of buying into the importance and value of the intervention and its components in relation to other existing practices (*legitimation*—secondary construct 7). Prior to implementation, stakeholders at the implementing organisation established the legitimacy of the intervention by relying on literature in the field about effective interventions along with pragmatic considerations regarding its applicability in real life settings. During roll out, seeking and utilising an official mandate to implement the intervention, ensuring rigorous oversight and monitoring of activity for quality, and following a systematic dissemination of intervention activities and related progress helped in establishing its legitimacy within communities. All these processes contributed to the *activation* (secondary construct 8) of the intervention and its components, which involved bringing forth the materials and means needed by stakeholders to operationalise the intervention in practice. At the community level, activation involved ensuring continued support from local governments and communicating a consistent message about the intervention and its progress. Within implementing organisations, a commitment towards sustained funding, developing systems and structures for continuity, liaising with different levels of partners, and troubleshooting ongoing barriers and challenges were important processes involved in activation.

#### 3.2.3 Collective action

Participants engaged in *collective action* (primary construct 7), which was purposive and directed towards intervention goals. This required investment of both intellectual and material effort. Firstly, enacting the intervention involved effort in the form of operationalising what needed to be done (*interactional workability*—secondary construct 9). A lot of groundwork had to be covered, prior to the start of the intervention. This involved seeking permissions from relevant authorities to implement the intervention; defining the intervention objectives and boundaries; roles and responsibilities of stakeholders involved; and developing an implementation plan. The implementation phase involved planning, coordination, managing logistics around delivery, setting up systems and structures at the organisation level. Embedding the intervention in existing structures and systems, building ownership and responsibility for the work and training and capacity building were some ways in which the intervention was operationalised by the participants. Effort was also invested in the way the intervention was mediated and understood by the network of people around it (*relational integration*—secondary construct 10). This further impacted the level of confidence and accountability stakeholders had in each other. Capacity building helped in developing expertise to deliver the activities as well as implement the materials within real life settings. Distribution and understanding of role boundaries not only helped in establishing rules governing distribution of work but also formal and informal expectations around the range of knowledge and expertise. An agreement regarding the validity of the work was built through processes such as communities of practice, supervision spaces, training around the collective philosophy and ethos of intervention delivery.

There were different ways in which the work itself was allocated to different stakeholders involved (*skill set workability*—secondary construct 11). This happened through three important means—policies and protocols related to the allocation of tasks, agreements regarding the necessary skills involved in delivering the tasks, and surveillance of the work based on this allocation. This was done through the development of protocols and intervention guides. The work was performed based on this allocation evidenced through the creation of specific governance and workgroups. At the community level, task allocation was implemented using communication strategies targeted towards specific stakeholder groups invested in the intervention. Importantly, this work was supported by host/implementing organisations (*contextual integration*—secondary construct 12) in many ways. Organisations provided support through two functions—execution and realisation. Execution involved procurement of resources (funding, capacity, and infrastructure) to implement activities; securing permissions to implement the intervention; and developing mechanisms for governance as well as evaluation. Implementing organisations helped realise the intervention and its components by providing capacity building and training support; lobbying with local governments and stakeholders; embedding the program within existing systems; and engaging stakeholders to ensure continued support for the intervention.

#### 3.2.4 Reflexive monitoring

Participants continuously engaged in a process of evaluating (formally and informally) their collective efforts, to arrive at an understanding of the intervention and its components (*reflexive monitoring*—primary construct 8). Prior to implementation, initial responses and impressions regarding the intervention were gathered through consultations with stakeholders. In the implementation phase, several mechanisms were developed to access this information (*systematisation*—secondary construct 13). Organisations maintained a data collection and monitoring system; asked questions about the delivery processes—what, how and why events happened; developed feedback mechanisms; conducted planned evaluation; used communities of practice for iterative problem solving and reflexive practice; and developed monitoring and oversight bodies. At the community level, systematisation involved keeping stakeholders updated about the intervention and its progress and conducting regular check ins with community members. These processes were methodological, formal mechanisms of obtaining information about the intervention and its effects. Appraisal of the intervention also occurred at a collective level (*communal appraisal*—secondary construct 14). Community stakeholders appraised the value of interventions through consultations prior to the delivery of an intervention. During implementation, this was facilitated through group meetings for iterative problem solving and documenting what happened on the ground, at an organisational level. Within the community, spaces for reflection were facilitated through consultations. Participants also appraised the intervention and its effects at an individual level (*individual appraisal*—secondary construct 15). This involved sharing and receiving updates about the intervention and its progress; receiving feedback from governing authorities; through conversations about what worked and what did not; mentoring and supervision spaces; and reflections during meetings. Communal and individual appraisals were experiential and unsystematic practices of judging the value and outcomes of a practice.

Finally, there were different ways in which participants modified their work in response to their appraisals (*reconfiguration*—secondary construct 16). This involved enacting changes in the implementation plan; changing the approach to the delivery of the intervention; and reassessing the scope of the intervention. In interventions with fidelity requirements, protocol deviations needed to be conveyed to relevant authorities. In some instances, feedback from community stakeholders was used to troubleshoot problems with the design and delivery of the intervention. In some contexts, support from local authorities was used to mitigate conflict.

### 3.3 What shifts in practices, organizational structures, interpersonal dynamics, and norms, can be observed as complex suicide prevention interventions are implemented, evolved, and integrated into various settings over time?

Although the focus of the study was not necessarily on examining *outcomes* (see Figure 1), a few reflections on the impact of the implementation efforts or outcomes were shared by participants (see Table 4). In terms of the practices that changed because of interventions being operationalised (*intervention performance*—primary construct 9), participants shared regarding the observed and enhanced capacity of a variety of stakeholders to implement the intervention as well as to offer support to people in suicidal crisis; streamlining of practices within existing systems; and onboarding of and partnership with local governments to ensure smooth delivery and maintenance of intervention activities. Narratives were also reflective of a few changes in the way people were organised and related to each other (*relational restructuring*—primary construct 10). All the activities geared towards mobilising and building capacity of community stakeholders enabled a sense of ownership for the program as well as a sense of responsibility for the well-being of the community. In some instances, the *success* of the program was linked to the contribution of local stakeholders and their willingness to engage. Instances of community stakeholders considering intervention activities as a part of their daily work, were also shared. The material practices and resources used to implement programs also helped in developing structures for problem-solving and examining the course of implementation.

**Table 4:** Overview of implementation outcomes.

Primary constructs- mechanisms	What are the practical effects of implementation at work?	Illustrative quotes
Intervention performance What practices have changed as the result of interventions and their components being operationalized, enacted, reproduced, over time and across settings?	Practices that have changed: capacity building of stakeholders in the intervention and its components; service sector integration; capacity to offer support; onboarding of and partnership with local governments; establishment of systems for various purposes.	“…so it really was around…Supporting the community to recognise and respond to suicide…suicidality, but also creating and supporting schools to do the same and supporting school students to do the same.” (Implementor 05)
Relational restructuring How have working with interventions and their components changed the ways people are organized and relate to each other?	A few things changed in the way people are organised and relate to each other—sense of ownership for the program; training of healthcare staff to promote more service sector integration; capacity building of stakeholders to intervene; intervention embedded into existing systems; support from local stakeholders; finding new ways to problem solve in a collaborative manner.	“And it then made us go back…And really talk again to workers and employers and industry and mental health experts who say…where are the solutions to these problems?…So, the program was designed in response to the problems that the industry faced. So it is actually about saying—How do you genuinely have something that is truly lived experience led truly industry led. And that was what came out of it.” (Lived experience advocate, 02)
Normative restructuring How have working with interventions and their components changed the norms, rules and resources that govern action?	A few changes in the norms, rules, resources that govern action are reported—renegotiation of roles of community stakeholders along with challenges to professional norms; changes in priorities of local governments; systems for more streamlined ways of functioning or data collection; synergies between departments; changes in ways of working—more ownership.	“One of the positive outcomes of that trial already is that we develop this surveillance system…And now that's a system that we designed, trialled it, published it and now WHO has taken it up and is now publishing a manual on it and putting it out as a system that other countries could follow.” (Leader 03).
Sustainment How have interventions and their components become incorporated in practice?	There are different ways in which the intervention becomes incorporated in practice: by creating allies and advocates of the work—building ownership; training and capacity building in new skills and in offering support; making the program a part of regular practice by embedding it within existing systems; partnership with local governments; learning new ways to problem solve.	“We had touch points with multiple services or multiple hospital networks. We could quite easily say…to the mental health nurse or the visiting psychiatrists that, hey, we're going to do this training in community now. You know…please let us know if it there's any adverse impacts…So we tried to manage that connection between what was happening in community and what was happening in tertiary, but also primary health as well.” (Implementor 04).

A few changes in norms, rules and resources that govern action (*normative restructuring*—primary construct 11) were also noted. In some contexts, there were shifts in understanding of roles. For example, in some contexts, community stakeholders attended gatekeeper trainings. This facilitated opportunities for them to work closely with healthcare systems and see themselves as an important partner in the intervention, holding responsibility for community well-being. Lobbying related activities pushed for changes in priorities towards mental health and suicide prevention within local governments; development of systems for more streamlined and representative data collection on suicides; and synergies between different local government departments responsible for health and well-being. A few glimpses of *sustainment* (primary construct 12) or the incorporation of the intervention into practice were gathered. Again, *success* was defined in terms of the extent to which the program becomes embedded in existing systems. This was specifically noted in intervention four, where the program became highly compatible within the industry setting. In other contexts, the stage for maintenance of the program was set by building skills among community stakeholders to perform tasks and activities, otherwise delivered, and managed with the support of the implementing organisation.

## 4 Discussion

In this study, NPT (1, 26), an implementation theory, was applied to understand how complex suicide prevention interventions are delivered, adopted and sustained within real life settings. A coding manual (27) defining domains and constructs described within NPT and its extensions, was used to understand the relationship between actions and their *mechanisms* (the things that people do and/or employ), *contexts* (the opportunity and transaction spaces that frame action) of implementation processes; and how these impact the *outcomes* (practical effects). To our knowledge, this is the first comprehensive application of the theory to examine *how* and *why* complex interventions in suicide prevention are effective.

### 4.1 Contexts are not passive entities

Unlike other models and frameworks that describe contextual determinants, NPT emphasises the role of *people* (individual and collective), their actions, material practices and resources that are involved in negotiating with the context. People constitute the context and implementation environment, actively or passively contributing to the delivery, adoption, and sustainment of interventions. Consequently, the theory encourages researchers to investigate the contributions of these people—the stakeholders, in addressing contextual influences to ensure the workability and integration of interventions.

Participants reflected on contextual determinants and *how* these impact various facets of the intervention. Across different interventions, contextual determinants could be thematically organised as macro, meso, and individual level factors influencing implementation processes. However, the nature of influence exerted by these determinants on the implementation environment seemed to vary across interventions. At the macro level, reflections on economic, legal, policy related factors impacting funding opportunities; policy engagement with suicide related issues; day to day operations; nature of stakeholder engagement; legality around suicides; and availability of data on suicides was reported by stakeholders from intervention four (Asia, a low- and middle-income setting). Other factors such as cultural beliefs around mental health and suicide, stigma; the complex etiology of suicide; availability of local evidence on suicide and suicide prevention were found to impact the design and approach to intervention delivery within this context. At the meso level, stakeholders from intervention one (Australasia, high income setting) reflected on issues related to staffing, size and availability of services; capacity building of staff and community stakeholders; time, and funding for implementation of activities across all components; building stakeholder engagement in the program which impacted the quality of implementation. At the individual level, individual and collective engagement in the program seemed to impact all interventions. These differences are unsurprising, considering the countries where these were implemented are vastly different.

We also identified reflections related to individual and collective intent, agency, and action. Participants expressed their agency by using contextual influences to modify the design and plan for delivery; made adaptations to the form and function of interventions and used characteristics of the implementation environment to suit intervention needs; leveraged the intervention environment to integrate the intervention into existing systems and practices. Despite differences in contextual influences, similarities were observed in patterns of negotiating with these influences. Across interventions two and six, there was an emphasis on adapting strategies and activities in response to the socio-political and economic environment and adjusting to the specificities of healthcare and social systems. Interventions three and four focused on a top down and a bottom-up approach to ensure engagement at all levels. Within these interventions, bringing stakeholders on board at all levels was a crucial negotiation strategy to integrate the intervention into existing systems. Across all interventions a common concern related to maintaining fidelity to the evidence-based intervention was expressed. Leveraging existing networks and relationships was found to be a common workaround to make an intervention workable within a specific context.

Findings from the study indicate that contexts often necessitate adjustments or adaptations to the initial design of an intervention. The dynamics within the context influence decisions regarding necessary actions and perceived gaps, consequently shaping different facets of complex interventions. According to extensions of the NPT (44, 45), implementation requires translational efforts. The outcome of implementation processes depends on interactions and negotiations between stakeholders and contexts. This means that understanding factors such as modifiability or plasticity of intervention components, the extent stakeholders have freedom over resource mobilisation and their own contributions, and the modifiability or elasticity of contexts (45) is crucial.

### 4.2 It is all about the work

The seminal work on realist evaluation (25) introduced a new approach to understanding how and why social programs work (or don't work). The approach is grounded in the philosophy of realism with an emphasis on identifying and understanding *mechanisms* that generate outcomes. Through a focus on program mechanisms we can transition from asking, “whether a program works to understanding what it is about a program that makes it work” (25; p. 5). Such an understanding departs from causation focused approaches that examine associated variables and correlates; to looking at explaining *how* the association itself comes about. In this study, an attempt was made to explore and examine generative mechanisms, responsible for implementation outcomes. The findings helped understand the implications of human potential, capabilities, and contributions in making programs work.

As mentioned, the focus of NPT is on the *work* involved in implementing programs. Four mechanisms of change were examined—(1) *what* is the work (coherence building); (2) *who* does the work (cognitive participation); (3) *how* is the work done (collective action); (4) *how* is the work *understood* (reflexive monitoring). Importantly, these mechanisms are dynamic and contingent (1, 26). The concurrent nature of activities associated with these mechanisms and their emergent production and reproduction over time was evident in the data (Table 3). Participant narratives not only highlighted the processes involved in implementing complex suicide prevention interventions but also provided insights into those who delivered the activities (implementing organisation, community stakeholders); the people involved in doing the action targeted (implementing organisation, community stakeholders); those who were targeted for change (community stakeholders) (46).

**Figure 2:**
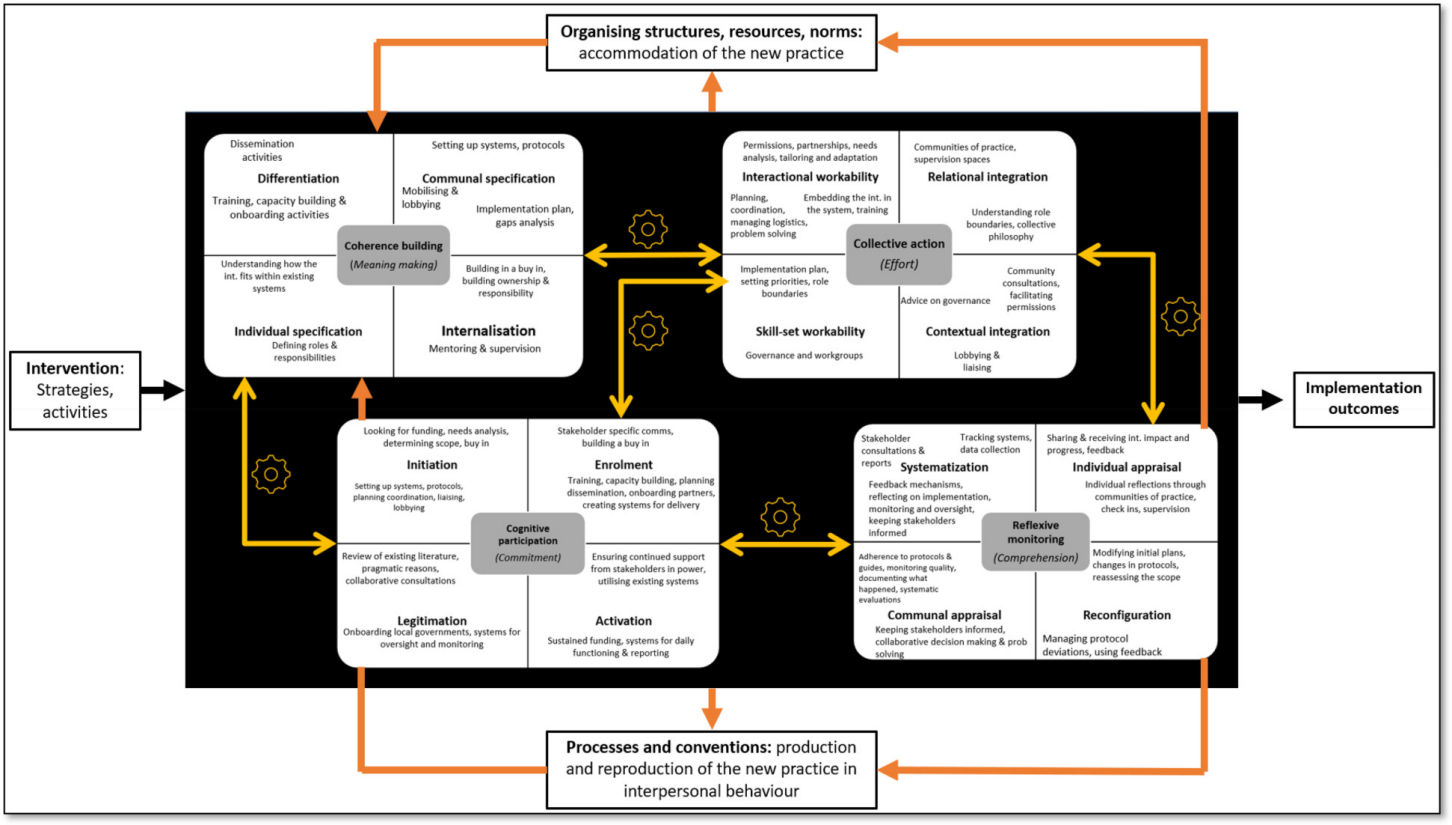
Illuminating the black box of complex suicide prevention interventions: context, mechanisms and outcomes. Conceptual model of Normalisation Process Theory (NPT): four constructs situated in a social and organisational context. Adapted from Vis et al. (48). This image has been adapted from an open-access article distributed under the terms of the Creative Commons Attribution License (https://creativecommons.org/licenses/by/4.0/).

The narratives allowed inferences to be drawn about processes at the organisational level—such as organizational changes and accommodations to implement interventions, and at the community level, regarding how interventions were operationalized in practice. It is not accurate to assume that the implementing organisation was solely responsible for delivering the intervention, while the community passively received the intervention. The reality was much more complex. While the implementing organisation was not the primary target of change, there was an ongoing negotiation process within both organisations and communities to accommodate the intervention. Integrating a new practice involved more than just following external directions (for communities); it required a continuous feedback loop between the two parties. For example, in doing the work (interactional workability), and individually and collectively reflecting (reflexive monitoring) on the effects of the work, community stakeholders would offer feedback to the implementing organisation about what happened on the ground. This would prompt changes in the approach to delivery (reconfiguration); in the work itself (interactional workability) and the confidence to do the work (relational integration) within organisations and communities. This further helped community stakeholders to enrol in the intervention and view it as being legitimate (cognitive participation). Hence it is evident that mechanisms can operate across different levels in a cascading sequence, where a change in a factor at one level leads to a change in a factor at another level, ultimately influencing outcomes (47) (see Figure 2).

Activities and strategies employed also varied across different phases of implementation, characterised by distinct goals and objectives as well as the nature of determinant factors impinging on implementation (49). The narratives indicated two phases—(1) pre-implementation phase: involving exploration and preparation prior to the roll out of activities on the ground; and (2) implementation phase: involving efforts towards adoption, delivery, and sustainment of interventions. In the pre-implementation phase, strategies for stakeholder onboarding and needs assessment at the community level and organisational readiness were commonly reported. Such strategies served the function of making sense of the intervention and understand roles, responsibilities, and tasks. In the implementation phase, several strategies related to training, resource allocation, delivery of activities, and developing feedback loops and monitoring systems, were employed. These strategies served the function of enabling stakeholders to do the work and reflect on their work. This further helped stakeholders discern what the intervention entailed, and reflections related to their own contributions. Furthermore, using the Expert Recommendations for Implementing Change (ERIC) taxonomy of implementation strategies (50), clusters of strategies could also be identified. The most common implementation strategies were *evaluative and iterative strategies* aimed at understanding needs; developing implementation plans; assessing various facets of the implementation process; engaging in monitoring and supervision. *Developing stakeholder interrelationships* and *engaging consumers* were other important clusters of strategies employed. This involved collaborative participation; mobilising stakeholder groups; using communication and dissemination; regular contact and keeping stakeholders informed. These findings were foreseeable and aligned with findings from reviews related to the use of implementation strategies in suicide prevention (51, 52).

Patterns were also observed in activities and strategies used by participants in implementing complex suicide prevention interventions. Interestingly, although there were similarities in activities and strategies across the four primary constructs, the underlying mechanisms differed. Simply put, the function of these mechanisms varied depending on phases of implementation and the socio-ecological level at which they were applied. For example, onboarding and training activities for staff within implementing organisations were common across all interventions. These activities served a dual purpose of helping staff differentiate (coherence building) between the intervention and current ways of functioning; and develop the capacity to initiate (cognitive participation) intervention activities within real life settings. Training and capacity building was also a common strategy for stakeholder engagement within communities. This served the function of helping community members differentiate the intervention; understand what was required of them individually (coherence building); initiate activities related to the intervention; do the work involved and build their sense of confidence to do the work (collective action). Similarly monitoring and oversight strategies used multiple mechanisms of change such as helping organisations internalise what the intervention entailed (coherence building); enrol staff in the activities, provide legitimation to the intervention (cognitive participation); help access information about the intervention and its effects, and reflect on its progress collectively and individually (reflexive monitoring). The same strategy served the function of ensuring legitimacy of the intervention and reflect on the intervention and its progress within communities. Analysing data from these vantage points helped understand the differences as well as the complexity of interactions between mechanisms across levels of the social ecology and phases of implementation.

### 4.3 Illuminating the black box

Figure 2 illustrates a dynamic process wherein delivery, adoption, and sustainment of an intervention (comprising several components) is influenced by interrelationships between several factors operating at different levels. The process begins when a social program (25) or intervention (complex interventions in this study) is introduced into the social system. A social system is a network of organized and changing relationships. These relationships create a structure comprising of agents or stakeholders (individuals or groups) who interact with one another. Through these interactions, information and other resources are exchanged between stakeholders (44). In this figure, the social system is represented through organising structures, norms, processes, and conventions. This forms the structural conditions within which mechanisms operate—unfolding processes over time that bring about or prevent some change (53). The intervention comprises strategies and activities which need to be operationalised and delivered. The process of implementation is deliberate and aims to operationalise new or modified practices. These actions are institutionally sanctioned (implementation organisation and the local authorities) and are performed by stakeholders involved (20). In this process, stakeholders (individuals and groups) work together to use and share material and cultural resources (1). This requires consent, cooperation, and expertise of those involved. Efforts (actions undertaken by stakeholders) towards delivery, adoption and sustainment of interventions activates mechanisms. A close look at mechanisms helps in understanding how stakeholders act on their circumstances (existing structures) and try to shape to shape them, to operationalise an intervention (44).

In this study, we endeavoured to illuminate the *black box* of complex suicide preventions by examining the underlying mechanisms of and factors associated with change. Figure 2 attempts at illuminating the internal workings of complex suicide prevention interventions. The two sets of arrows (yellow and orange) represent different feedback loops in the process of implementing complex interventions. Mechanisms comprised of primary and secondary constructs are emplaced inside the black box. The yellow arrows represent interdependencies between these mechanisms. As can be noted, these relationships are not linear. The loop emphasises the dynamic and iterative nature of implementing interventions, where strategies and activities are continually refined based on how mechanisms feed into one another (as illustrated previously). The orange arrows represent another kind of feedback loop—one that emphasises the continual adjustment and reinforcement of the broader organisational and normative context in accommodating and sustaining a new practice/intervention. Simply put, continuous investments by stakeholders, related to meaning, commitment, effort and comprehension carry forward in time and space; and sustain the integration of a practice in its social contexts (1, 26). This work and investment contributes to intervention *success* or *failures* (outcomes). In summary, the diagram underscores the complexity and iterative nature of implementing complex suicide prevention interventions. It highlights the need for understanding comprehensive strategies, continuous feedback loops, and constant accommodation and negotiation of the social system, to achieve successful and sustained implementation outcomes.

## 5 Implications

The objective of the study was twofold: to enhance the understanding of processes involved in the delivery, adoption, and sustainment of complex suicide prevention interventions; and to advance the application of the NPT to address real-world issues. Important questions were raised. The first question aimed to understand the dynamics of human agency under conditions of constraint (45). Implementation was understood through the lens of feedback loops, adaptive mechanisms, and compromises, indicating the non-linear and dynamic nature of processes. The findings of the study highlight a need for a shift in perspective around how contexts are understood, conceived, and utilised in implementation efforts. Hawe et al. (54) suggest a few ways in which this shift can occur—(1) thinking about and factoring in relationships among people or agencies as part of the context which can help understand why interventions work better in one setting over another; (2) standardising interventions across sites by function rather than form, which may allow for adaptation to context while maintaining fidelity; and (3) allowing for longer time frames to observe the changes occurring within dynamic systems as a result of human activity and engagement. This closely relates to the idea of negotiating and being intentional about what constitutes the intervention of interest and what constitutes context. Observing and examining these interactions over time can provide valuable insights into domains requiring adaptation and identify mechanisms of how to achieve this change (19).

The second question aimed to explore the underlying mechanisms which influence implementation outcomes of complex suicide prevention interventions. In recent years, although there have been efforts to understand what interventions are effective, the questions of *how* and *why* they work have been overlooked. This study leveraged the invaluable experiences of key stakeholders who have actively addressed these crucial questions in their day-to-day practice. Without consolidating and analysing this experience and knowledge, suicide prevention initiatives risk reinventing the wheel. Understanding mechanisms by which implementation strategies address contextual barriers to change is important. Unless this gap is addressed, practical guidance regarding which implementation strategies to use will remain inconclusive (55).

A step toward understanding effective, feasible implementation strategies involves identification of multilevel mechanisms through which these strategies influence implementation outcomes (47). In implementation science, considerable efforts have been made to understand mechanisms of change within health-related interventions. Despite these efforts, substantial conceptual, methodological and measurement issues have been noted in advancing mechanisms-based implementation research (56, 57). Qualitative research has been highlighted as important means to inform measurement and theory development. Van Belle et al. (58) summarise critical reasons for using more theory driven approaches. Firstly, theories are well suited to demonstrate the interplay between policy, program, context, causal mechanisms, actors or stakeholders and outcomes, and hence are “complexity consistent” (59; p. 405). Secondly, it has been argued that theories provide pragmatic tools to understand “the nuts and bolts” of interventions and in the process demonstrate how theories are built and applied. This further helps advance and refine theories. Thirdly, application of theories helps mobilisation of ideas and encourages us to look at complex problems through an interdisciplinary lens. Evidently, there are challenges in the use and application of theories such as resources involved in operationalising and testing them, especially in and low- and middle-income settings. Conversely, ensuring effective and comprehensive use of funds is even more crucial, as waste is particularly damaging in these contexts.

A few studies within suicide prevention have also attempted to understand the operationalisation of practice in real life settings using the NPT (60, 61). Decades of research into the NPT and its constructs has prompted diverse investigations regarding the work people do to achieve specific goals. A series of theoretical and empirical studies about how stakeholders take up healthcare work and embed these in their daily lives have been conducted (62, 63). There are also models and measures of the cumulative complexity of chronic conditions (64) and analysis of patient experiences (45). This has also led to the development of theories related to collaborative organisations of practice (65). Lessons from the application of NPT in these studies can be applied to suicide prevention research.

## 6 Limitations

Some limitations need to be underscored. The NPT was applied retrospectively to analyse qualitative data. Participants were not directly asked about the mechanistic processes outlined in the NPT coding manual. Therefore, insight into these mechanisms is an emergent outcome of the inquiry. The study did not involve a formal evaluation of the interventions included. The purpose was to understand experiences of implementing complex suicide prevention interventions. Although this yielded a reflection on strategies and activities employed to enact interventions; we did not consider which of these strategies were effective (or not). This is an area that requires further exploration. The findings suggest that the activities and strategies were implemented without any challenges. However, this was not the case. The challenges (42) and barriers (43) in implementing these interventions have been summarised elsewhere.

The study also focused on an aggregate of experiences of different stakeholders, across different interventions and country contexts. This was done to develop a general theory around how stakeholders work to implement interventions and what contributes to certain outcomes. As a result, nuances (stakeholder, intervention, country context) may have been missed. Potential differences in the number of participants across stakeholder groups represented in the sample and their capacities to provide insight into underlying mechanisms, may have impacted a comprehensive understanding of the mechanistic processes/factors. Findings were inferred from retrospective accounts of participants. Hence, inferences were drawn from these reflections could be compromised due to challenges in recall. Finally, the number of informants per intervention were relatively limited.

## 7 Conclusion

This is a novel study using NPT to understand how and why complex interventions work in real life settings. Data from key international stakeholders was used to develop a general theory of implementation of complex suicide prevention interventions. The data reflected continuous investments by stakeholders, related to meaning, commitment, effort and comprehension that carry forward in time and space; and sustain the integration of a practice in its social contexts. The findings offer valuable insights for the design and evaluation of suicide prevention interventions, providing a blueprint to inform practice and predict outcomes. There is a need for a paradigm shift towards the use of more theory based and informed approaches to understand causal links between implementation strategies, context, mechanisms, and outcomes.

## Data Availability

The original contributions presented in the study are included in the article/Supplementary Material, further inquiries can be directed to the corresponding author.
