# Supplementary material for: Illuminating the “black box” of complex suicide prevention interventions: towards a theory of implementation using the normalisation process theory
Source: Front Health Serv. 2025 Aug 7;5:1473682. doi: 10.3389/frhs.2025.1473682 (PMC12367768; doi:10.3389/frhs.2025.1473682)
Supplement: Supplementary file 2 [file Table1.docx]

Supplementary Material

**Supplementary material 1**

**Interview Guide**

*Hello and welcome. I am _________ (name), and work as a ________(position). I am interested in _____________. I will be guiding this discussion today. As mentioned previously, you have been invited to participate in this study because of your experience of implementing complex suicide prevention interventions. Several definitions of complex interventions exist- we have recently published a paper on this fluidity of terms and definitions. Within the scope of this study a complex intervention is a multilevel, multicomponent, multimodal, systemic, multimodal,* and *integrated* intervention.

*In today’s discussion, we will explore your experiences of implementing __________ (name of the intervention) specifically and also attempt to generally understand what makes complex interventions* ***work*** *in real life settings. This information is invaluable as it will help address important questions about what works, why, for whom and in what context.*

*This discussion will take approximately 1.5-2 hours. Please feel free to share the breadth of your experiences since it will be kept confidential. Your responses will be deidentified as we are interested in understanding an aggregate of experiences. Please feel free to let me know when you need to take a break and/or stop the interview if you feel uncomfortable.*

*This discussion will be recorded. If you are uncomfortable with a video recording, you can switch off your camera such that only your voice is recorded.*

*Once the interview is complete, a transcript will be shared for your reference. You will have a chance to look through your responses and make modifications if necessary.*

*Do you consent to participating in this interview?*

*Before I begin, I need to ask if you are you having/have had thoughts of suicide now or in the past 6 months?*

Instructions for the interviewer:

- Approach the interview as a discussion such that it seems like a conversation rather than a question-answer process.
- The guide is divided into 3 sections – starting with introductory questions which set the stage for the interview process; exploratory questions which aim to explore the depth of their experiences; and exit questions which aim to conclude the discussion.
- Ask each question individually, one after another.
- This is an open-ended conversation. Hence, keep an eye out on the questions which have been answered/addressed to avoid repetition.
- Some questions comprise a main question (highlighted in bold) and sub-questions as probes and prompts. When the participant is unclear or feels stuck about how to respond to a question, use probes.
- Encourage participants to share examples to help understand their experiences better.
- Pay attention to the verbal and non-verbal cues and reflect them in your conversation with the participant. For example – “you look excited about this. Could you share more about what makes you excited?”

**Demographics**

- Name
- Email ID
- Gender

**Engagement/introductory questions**

- Can you tell me about yourself and your work?
- What interests you about suicide research and prevention?
- How did you come to be involved with ____________ (name of the intervention)?
- Could you describe what your role was/is within the intervention/project?

**Exploratory questions**

For leaders:

- Could you tell me more about the design of the project/intervention? How did you conceive the intervention?
- What was your theory of change? How did you plan to achieve the outcomes outlined?
- What was your strategy for implementation? (planning, recruitment, community engagement, implementation plan).
- Could you outline the overall processes involved?
- How did you monitor progress of activities?
- How did you conceive of the evaluation plan? What did the evaluation plan entail?
- In retrospect, what was the experience of leading/managing such an intervention like for you? What stood out the most (key takeaways)? What is different from other work experiences you have had?
- What were your biggest challenges? (individual, organisational, community setting)
- How did you address these challenges? What was helpful/unhelpful? Do some challenges continue to remain unaddressed?
- What were your most important learnings? Have you documented these learnings?

For implementation practitioners

- How did you come to know about the intervention/program/project? Could you tell me more the recruitment process and your roles and responsibilities?
- What was your understanding of the intervention and its relevance?
- To what extent did you feel involved in the implementation of the project? Could you give examples?
- What were your day-to-day tasks and activities? What were the general categories of tasks over a week/over a month?
- What was your strategy for executing these tasks and activities?
- What were some examples of organisational processes followed? How did you engage with the community?
- How was quality monitored?
- In retrospect, what was the experience of working as part of such an intervention like for you? What stood out the most (key takeaways)? What is different from other work experiences you have had?
- What were your biggest challenges? (individual, organisational, community setting)
- How did you address these challenges? What was helpful/unhelpful? Do some challenges continue to remain unaddressed?
- What were your most important learnings? Have you documented these learnings?

For lived experience experts

- How did you come to know about the intervention/program/project? Could you tell me more the recruitment process and your roles and responsibilities?
- What was your understanding of the intervention and its relevance?
- How was participation in co-design for you? What stood out the most?
- To what extent did you feel involved in the implementation of the project? Could you give examples?
- What was your strategy for executing these tasks and activities?
- What were some examples of organisational processes followed?
- How was quality monitored?
- In retrospect, what was the experience of working as part of such an intervention like for you? What stood out the most (key takeaways)? What is different from other work experiences you have had?
- What were your biggest challenges? (individual, organisational, community setting)
- How did you address these challenges? What was helpful/unhelpful? Do some challenges continue to remain unaddressed?
- What were your most important learnings? Have you documented these learnings?

***Exit questions***

(Applicable to all)

- What do you think is needed to prevent suicidal behaviour?
- What do you think is the relevance of complex suicide prevention interventions in preventing suicidal behaviour? What is the future of these interventions?
- Based on your experiences and learnings, what is most needed to better understand the implementation of complex suicide prevention interventions? How do we improve the practice and science of complex interventions in suicide research (from each stakeholders’ perspective)?
- What do you think is the way forward?
- Would you like to add any other information that has not been mentioned yet?

**Supplementary Material 2**

**Participant Information Sheet**

**Title: Illuminating the black box of complex suicide prevention interventions: Harnessing implementation science approaches**

**Research Information sheet: In-Depth Interviews**

**Purpose of the research**

The aim of this study is to understand experiences of individuals involved in the implementation of large-scale, complex suicide prevention efforts. The objective is to understand what is involved in making these interventions **work** in a real-world setting. This research forms part of a PhD being conducted by Ms. Sadhvi Krishnamoorthy under the supervision of Professor Kairi Kolves, Dr. Victoria Ross and Dr. Gregory Armstrong

**What does it involve?**

Participation in this research project involves an in-depth interview which will be conducted through an online platform - Microsoft Teams. During this interview you will be invited to share your experiences of being part of design and implementation of large scale suicide prevention efforts. The interview will be conducted in English and will take approximately **1.5-2 hours**. However, if you are more comfortable expressing in a language other than English, we can discuss the kind of support measures you may need to express yourself in a language of your choice and preference. It will be scheduled at a time most convenient to you.

**Who can be involved?**

You have been invited to participate in this study because you have had experience of implementing complex suicide prevention interventions. You are being interviewed because:

- You are/have been a Principal and chief investigator, project directors, senior research fellows, heads – individuals who have had experience of leading complex suicide prevention interventions.
- You have past or current experience in managing overall conduct of complex interventions

**OR/AND**

- You are/have been a project team member, project manager, day to day implementation practitioner – an individual who has had the responsibility of ensuring interventions are implemented as per protocol.
- You have past or current experience of implementing complex suicide prevention interventions

**OR/AND**

- You are a person with lived experience of suicide, bereaved by suicide, caregiver of person with lived experience of suicide who has participated in co-design in paid academic role/employee/consultant/advisor.
- You draw on your lived experience of suicide to ensure the needs of people with lived experience are well represented in the design and implementation suicide prevention efforts.
- You have no current suicidal behaviour, which means no current intentional ideation or planning - not less than 6 months prior to the interview date and/or a person bereaved by suicide not less than 6 months before participating in the study.

**What are the benefits of participating in this study?**

There are no direct benefits to you for participating in the study. However, your participation is invaluable to the study. Your experiences will help develop a better understanding of how suicide prevention efforts are implemented in real-life settings. These experiences can potentially guide and inform future efforts in suicide research. In practice, this will benefit those with lived and living experience of suicide, for whom these efforts are designed. In research, your participation will help understand and refine our understanding of what is required to ensure public health impact. Findings from the study will also help inform policy makers to focus on efforts that have proven to create an impact. Overall, your participation can help improve research and service provision in suicide prevention.

**What are the risks?**

There is no significant risk associated with participating in this study. When you talk about your past/current experiences of involvement with a suicide prevention program/intervention, it is possible that you are reminded of unpleasant experiences (along with pleasant experiences). You may also be reminded of situations/people/places that created challenges for you. These experiences may create some distress. The interviewer is trained in active listening and empathy and help you with these feelings. After the interview is finished, you may want the support of your family and friends. You can also access support from the support services enlisted at the end of this document.

Please note that while we are aware that different individuals may have a personal lived experience of suicide, you will not be asked to disclose information pertaining to your lived experience.

**Your confidentiality**

Any information that you disclose will be confidential. However, the results of the study will be reported in an academic thesis and may also be disseminated via journal articles and/or conference presentations. The results will be presented in a way that will not identify you or your relative/friend. The data from this study and the audio recording will be electronically stored on a password protected computer. Identifiable data will be stored for 5 years from the publication of results, after which time all identifiable data will be destroyed.

**Your participation is voluntary**

Participation in the study is optional. It is expected that people wishing to participate should be prepared to engage in an interview that lasts up to 1.5-2 hours. Your participation in this project is voluntary, and you are free to withdraw at any time, without explanation or penalty.

**Feedback to you**

At the end of interview, a transcript detailing our conversation will be sent to you. You are free to review and edit your responses and contribution.

You can also request access to a plain language summary of research results using the following email address: [sadhvi.krishnamoorthy@griffithuni.edu.au](mailto:sadhvi.krishnamoorthy@griffithuni.edu.au)

**Questions / further information**

If you have any questions, please do not hesitate to contact:

**Interviewer**

Sadhvi Krishnamoorthy

Australian Institute for Suicide Research and Prevention (AISRAP)

Griffith University

Phone: +61 420614267

**Email:** [sadhvi.krishnamoorthy@griffithuni.edu.au](mailto:sadhvi.krishnamoorthy@griffithuni.edu.au)

**Researchers involved:**

Kairi Kõlves, Professor, AISRAP, School of Applied Psychology, Griffith University

Victoria Ross, Senior Research Fellow, AISRAP, School of Applied Psychology, Griffith University

Gregory Armstrong, Senior Research Fellow, Melbourne School of Population Health, University of Melbourne

Sadhvi Krishnamoorthy, PhD Candidate, AISRAP, School of Applied Psychology, Griffith University

**The ethical conduct of this research**

This project has been approved by the Griffith University Human Research Ethics Committee - GU ref no: 2022/286

Griffith University conducts research in accordance with the *National Statement on Ethical Conduct in Human Research.* If you have any concerns or complaints about the ethical conduct of this research project, please contact:

Senior Manager

Research Ethics and Integrity

Griffith University

Ph: 07 3735 4375

Email: [research-ethics@griffith.edu.au](mailto:research-ethics@griffith.edu.au)

You may easily identify this project by GU Ref No: 2022/286

**Privacy Statement**

The conduct of this research involves the collection, access, storage and/or use of your identified personal information. The information collected is confidential and will not be disclosed to third parties without your consent, except to meet government, legal or other regulatory authority requirements. A de-identified copy of this data may be used for other research purposes - research results will be reported in an academic thesis and may also be disseminated via journal articles and/or conference presentations.

However, your anonymity will at all times be safeguarded.

For further information consult the [Masked for Blind review]

**Support services**

If participation in this study makes you feel sad or distressed in any way, please do not hesitate to reach out for help. The following support can be accessed in __________ (this was based on the country context)
